# Becomings beyond the ideal: women's fitness and digital subjectivities in the Douban women's fitness community

**DOI:** 10.3389/fspor.2026.1786979

**Published:** 2026-05-01

**Authors:** Siqi Fan, Kohei Kawashima

**Affiliations:** 1 Graduate School of Sport Sciences, Waseda University, Tokyo, Japan; 2 Faculty of Sport Sciences, Waseda University, Tokyo, Japan

**Keywords:** Chinese online community, feminist counterpublic, feminist new materialism, fitness culture, women's fitness

## Abstract

As women's fitness gains cultural visibility in China, it has become a key site for contesting dominant baishouyou beauty ideals (fair-skinned, slim, youthful). Grounded in Feminist New Materialism (FNM), this paper examines *Women Fitness*, a women-only Douban community, as a feminist counterpublic that reconfigures fitness from a neoliberal “self as project” into a collective infrastructure of care and resistance. Using a mixed-methods design that combines digital ethnography, topic modeling, sentiment analysis, and engagement metrics, the findings reveal how participatory governance, affective circulation, and embodied multiplicity collectively enable feminist becomings. It formalizes moderation practices to support grassroots resistance to pseudoscience, body ideals, and influencer logics, while its affective metabolism transforms negative affect into collective affirmation. By centering lived materiality, the community fosters an embodied multiplicity that disrupts normative narratives of progress. While entangled in structural inequalities, it materializes posthuman feminist subjectivities, offering a blueprint for digital health activism by prioritizing relational becoming over individualistic self-optimization.

## Introduction

The rise of women's fitness icons such as Zhang Weili and Jia Ling marks a shift in China, where more women are using physical training to challenge “baishouyou” ideals (1, 2). Yet fitness remains a contested domain, shaped by hegemonic masculinity: masculine traits are positioned as the norm, while women’s fitness is frequently marginalized to body projects that prioritize hyper-visibility and neoliberal self-surveillance (3–5).

While foundational feminist scholars have established the body as a site of self-discipline (6, 7), digital fitness culture necessitates an updated lens. Current scholarship has begun to explore how digital platforms facilitate postfeminist sensibilities, in which empowerment is sold through consumer capitalism (5, 8). Mainstream women's fitness culture in China reflects this neoliberal regime, where women's bodies are positioned as individual projects of optimization, requiring constant regulation and enhancement (9–12). However, most existing literature focuses on Western, public-facing visual platforms such as Instagram and TikTok. An empirical gap remains regarding semi-private, text-centered digital communities in non-Western contexts, which may resist the drawbacks commonly associated with mainstream platforms, as noted above.

This study addresses this gap by analyzing *Women Fitness*, a women-only digital community on the Chinese platform Douban. Unlike influencer-driven mainstream fitness cultures, this community redefines fitness in relational, pluralistic, and processual terms, centering sustainability, fluctuation, and care. To analyze this, this study draws on FNM to conceptualize *Women Fitness* as an assemblage of governance, affect, bodies, and subjectivities and examines material–affective entanglement (13–16), alongside Intersectionality, to maintain attention to structural inequalities (17, 18).

Methodologically, this study is the first to operationalize FNM concepts through digital ethnography combined with computational analysis, drawing on a database of 5,626 posts and 114,019 comments. In computational terms, affective circulation is measured as sentiment density via sentiment analysis, while the stickiness of non-aesthetic thematic clusters is examined via topic modeling. Material–affective entanglement is identified through the co-occurrence of physical experiences and digital tracking data, analyzed via topic modeling and engagement metrics. In digital ethnography, the grassroots sustainability threshold is reflected in the ways members' engagement becomes sedimented into community guidelines. This study asks the following questions: (1) What dominant discourses emerge in *Women Fitness*, and what values do they reflect? (2) How do emotions circulate and shape community interactions? (3) What content attracts the most engagement, and why? (4) What tensions and possibilities arise in cultivating feminist counterpublics within digital fitness culture?

## Literature review

Over the last two decades, online communities centered on women's embodied practices have become key cultural spaces in which norms of femininity, health, and empowerment are both performed and contested (19, 20). Platforms like Instagram and TikTok blur the boundaries between self-care and self-surveillance, offering spaces where identities are as much curated as they are constructed (21). Central to this terrain is Fitspiration (Fitspo), a seductive blend of discipline, health, and aesthetics (22, 23). While often framed as promoting health, Fitspo reproduces bodily ideals centered on toned, lean, muscular, able-bodied, and often white femininity (24–28). Tracing the evolution from Thinspiration (Thispo) to Fitspo, alongside the rise of counter-movements such as Body Positivity (BoPo) and Body Functionality, reveals how bodily ideals are simultaneously challenged and reinscribed—shaping subjectivity as much as they reflect it (29, 30).

### Thinspiration: idealizing frailty

Rooted in pro-eating disorder subcultures, Thinspo communities glorify skeletal thinness using hypersexualized, dismembered imagery captioned with slogans that equate disordered eating with control and attractiveness (22, 24, 25, 31, 32). Tags like “bonespo^1^” and “skinny” normalize pathology, further reinforced by peer validation (22, 32, 33). Exposure to such content is linked to body dissatisfaction, self-objectification, and disordered eating, particularly among adolescent girls and young women (34–36). While Social Comparison Theory, Objectification Theory, and Social Cognitive Theory help explain these influences, they often overlook the collective dynamics that shape identity within these communities.

### Fitspiration: rebranding the ideal

Fitspo rebrands bodily discipline through wellness, featuring toned bodies and slogans such as “Strong beats skinny every time” (22, 27). However, it continues to emphasize appearance, sexualized aesthetics, and moralized self-discipline, with limited evidence of health impact. Exposure to Fitspo imagery has been shown to increase appearance-based comparisons and body dissatisfaction, without translating into improved exercise behavior. It reconfigures harmful ideals in more socially acceptable forms, framing aesthetic control and self-discipline as moral virtue (20, 23, 37–39). Despite rejecting Thinspo's frailty ideal, Fitspo aligns with neoliberal sensibilities that equate empowerment with aesthetic achievement and personal responsibility, functioning as a rearticulation of patriarchal norms in the language of choice and agency (24, 25, 27, 33).

### Critical pushbacks: body positivity and body functionality

BoPo promotes self-acceptance and diverse beauty; however, it often centers visibility, tethering women's value to “being beautiful” in more inclusive, natural, or authentic ways (35, 40, 41). In contrast, the Body Functionality approach reorients value away from how bodies look toward what bodies do (42, 43). However, it risks reinforcing ableist and productive norms (44). Both BoPo and Body Functionality reveal tensions between visibility, normativity, and feminist resistance. Their potential lies not in promoting one ideal over another but in disrupting aesthetic hierarchies and centering structural inclusivity (45, 46). Still, they remain entangled in neoliberal logics, where health and empowerment are framed as matters of individual responsibility and consumer practice (29, 47–49).

### New possibilities: beyond norms, toward becoming

Feminist New Materialism (FNM) destabilizes ideal-centric embodiment by emphasizing affective and material entanglements among bodies, technologies, and environments (20, 50, 51). However, recent Chinese studies has largely focused on criticizing neoliberal discourses that position self-discipline as a moral duty (9–12), reproducing paradigms already problematized in Western literature. This study seeks to move beyond previous critiques by integrating FNM with intersectionality, spotlighting both affect and matter, as well as unequal power across various axes.

## Theoretical framework

Poststructuralist feminism, particularly Butler's (52) theory of gender performativity, challenged essentialist concepts by framing gender as a regulated repetition rather than a fixed identity. However, such discourse-centered models can marginalize the material and affective aspects of embodiment (53–56). FNM builds on poststructuralism but shifts the focus toward ontology, affect, and matter. Drawing on Deleuze and Guattari's (57) concepts of becoming and assemblage, FNM theorists reconfigure subjectivity as emergent, relational, and shaped through entanglements with non-human forces, affects, technologies, and environments (14–16, 58). This framework allows us to explore how subjectivities are not only socially constructed but also materially enacted through dynamic material-discursive processes (15, 55, 59, 60). Applied here, FNM foregrounds how feminist subjectivities emerge through intra-actions between members, discourses, affects, and forms of knowledge. However, FNM has been critiqued for obscuring histories of power (61–63). To address this limitation, this study integrates intersectionality, retaining attention to power, history, and accountability.

## Methodology

### Ethical considerations

Membership is required to post and comment but not to view content. Therefore, there is no posted content behind a membership gate. Formal IRB review was not required, as this study analyzes pre-existing online content that is publicly viewable and involves no interaction with or recruitment of members (64). Following the AoIR Ethics 3.0 guidance (65), we hashed member identifiers and translated quotations into English to prevent “reverse searching” and re-identification (66), thereby maintaining the contextual integrity of the data while minimizing potential harm. Collected strictly for non-commercial academic research, the data were handled within a secure environment to ensure they neither compete with nor redistribute proprietary platform interests, adhering to principles of fair use in digital research (67).

To further ensure integrity, inclusion and exclusion criteria were applied:

- Inclusion criteria: posts and the subsequent comment section.
- Exclusion criteria: non-textual multimodal content, such as emojis, memes, photographs, and videos; deleted or administrator-removed content; irrelevant information, including location names, references to animals, time phrases, pronouns, prepositions, conjunctions, particles, discourse markers, punctuation, formatting symbols, numerical noise, units, measurable terms, and kaomojis; and reposts originating outside *Women Fitness*.

### Platform affordances and community mechanics

Douban communities operate through a semi-private, forum-style interface that differs significantly from the algorithmic “For You” feeds of public platforms such as TikTok or X. Within *Women Fitness*, content visibility is primarily driven by a reverse-chronological “bumping” mechanism, whereby any new comment moves a thread to the top of the community's front page. This technical affordance means popularity is not determined by an algorithmic engine; instead, it emerges organically from interactional density. However, this organic surfacing is moderated by specific administrative affordances: pinned threads ensure that core guidelines remain permanently visible, while highlighted status provides long-term visibility to specific archives. By analyzing textual data within this moderated interface, we recognize that what appears to be popular and affective content is a co-production of member engagement and intentional moderation actions.

### Digital ethnography

This study adopts digital ethnography, recognizing the internet as embedded, embodied, and part of everyday life (68). One author joined *Women Fitness* in September 2024 after passing an entry screening focused on feminist and fitness literacy and remained a non-participatory observer. Systematic archival observation and archival capture were conducted from September 2024 to April 2025. During this period, one author conducted an in-depth review of 166 threads, including six pinned threads, the top 20 high-engagement threads, 50 early-stage posts, and all editorially highlighted threads within the community. Fieldnotes were recorded as a part of the results (Tables 1–3, 4), and detailed community guidelines, archived pinned threads, editorial highlights, and moderation logs were uploaded on OSF.

**Table 1:** Aesthetic posts in July 2022 (excerpts).

Date	Title
220707	Hey girls, can I slim down my thick arms by lifting dumbbells?
220710 220720	How can I lose fat efficiently? What kind of training will give me a slender, poised figure and a perfect waist-to-hip ratio?
220721	Feel like I'm stuck and not losing any fat.
220723	About leg hair.
220725	Sis! I wanna ask, can you really tone your ankles by exercising?
220725	Can you get a peach booty by working out at home?
220726 …	It's been three months of working out, and I've seen zero change in my abs. …

**Table 2:** Comments against body image anxiety in July 2022 (excerpts).

Date	Comments
220707	There is no such thing as localized fat reduction […] When you get thinner, it's your whole body that gets thinner. As for getting bulky, […], don't worry about it, you are barely liftting.
220710	Fitness progress is measured in long-term, just let it happen naturally. And please, don't skip dinner!
220720	The chance of having a high waist-to-hip ratio but also thin and slender limbs in real life is very low, it mostly depends on genetics, […] “Ideal” body is a definition of “sexy” shaped by patriarchal fantasies, which women have every right to reject and ignore.
220721	Your body fat is within a perfectly healthy range, there's no need to lose more. Super low body fat is not sustainable.
220723	There's nothing wrong with having hairy legs.
220725	Pretty sure you can't get slimmer ankles through training. It's just genetics.
220725 …	If you lose any more weight, you'll be nothing but bones. Your butt isn't the shape you want not because it's fatty, it's because you don't have enough muscles. …

**Table 3:** Unacceptable behaviors.

Category	Examples
Male presence and male gaze content	Male accounts, images with unblurred male figures, male validation
Advertising, promotion, and monetization	Advertisements, celebrity-related, or identifiable photos
Body ideals, anxiety, and shaming	A4 waist, 90-degree shoulders, peach booty; body shaming; low body fat obsession
Low-quality or redundant content	Repeated check-in threads, redundant questions already answered in FAQ
Inappropriate or irrelevant content	Vulgar, pornographic, or sexual; non-fitness content; personal insults
Disruptive or malicious behavior	Trolling accounts, DM harassment
Unsafe or misleading practices	Pseudoscientific claims, unsafe dieting, or unqualified medical advice

**Table 4:** Posts about lived bodily struggle (excerpts).

Title	Summary
Relationship with food	Criticizes rapid weight-loss diets as harmful and unsustainable, instead promoting exercise and a balanced, long-term approach to food and health
How can I get my mom to love exercising?	Concerned about her mother's physical and mental health and her resistance to exercise due to knee issues, the author received advice from members recommending gentle practices, social engagement with peers, and positive reinforcement to encourage adherence
Work out during period	The author summarized workout tips around the period. Rest for the first three to four days for better muscle recovery and fat metabolism. From day 4 to about three to four days after bleeding ends, focus only on strength/resistance training, mainly upper body at first, then lower body muscle groups. About 10 days after the period ends is the best phase for cardio
What can I do about Achilles tendonitis?	The author sought advice to reduce Achilles tendonitis. Members suggested frequent calf raises and stretches, keeping the ankle warm, wearing supportive shoes, and walking on tiptoes
Sisters, after exercising, my sleep quality has gotten worse. What should I do?	Experiencing poor sleep since starting late-afternoon strength training and brisk walking, the author received member advice to adjust workout time to earlier, allow adaptation, ensure proper nutrition, and consider magnesium or vitamin B supplements
Reflection on the Itaewon crowd crush	Responding to the Itaewon crowd crush, the author emphasized avoiding crowds, physical strength, and nutrition for survival. Members agreed that muscular robustness counters vulnerability and critiqued “slim” beauty ideals that discourage women from strength training
…	…

### Coding process

To complement qualitative insights, this study integrates three computational methods commonly used in digital culture research: topic modeling, sentiment analysis, and engagement analysis (69, 70). The dataset comprises a complete census of all accessible, member-generated posts and comments within *Women Fitness* (https://www.douban.com/group/733756/) between July 2022 to September 2024, as publicly accessible in the community as of 22 September 2024.

Quantitative data analysis was conducted in Python. To identify the thematic architecture of *Women Fitness* discourse, topic modeling was performed using BERTopic. The Sentence-Transformers model “sentence-transformers/all-mpnet-base-v2” was used to generate document embeddings, configured with minimum_topic_size=15. Dimensionality reduction was managed via UMAP, configured with metric="cosine', n_neighbors=8, n_components=8, min_dist=0.1, and random_state=42. Clustering was performed using HDBSCAN in the UMAP-reduced embedding space with the minimum_topic_size=15 (aligns with min_topic_size), metric=“euclidean,” cluster_selection_method = “eom,” and cluster_selection_epsilon=0.0. Chinese text preprocessing was conducted using NLPIR SDK 2024 prior to topic representation. The computational workload was optimized using GPU acceleration, processing batch_size=48 and automated mixed precision, and a maximum sequence length of 128 tokens. For validation, an inter-topic distance map was generated to evaluate topic separability and identify potential overlaps. The resulting topics are distributed across four quadrants without overlap, demonstrating high thematic distinctiveness and minimal redundancy.

Sentiment analysis was performed using the RoBERTa-large^2^ multilingual sentiment model (71). This model was selected for its superior performance compared to traditional machine learning classifiers and older word embedding techniques, achieving high levels of accuracy (72). Before scoring, data with empty token strings were removed. Texts exceeding 500 characters were segmented using a sentence-aware sliding window strategy (≤300 characters) to maintain semantic coherence. The model outputs were mapped to a continuous score within the [−1, 1] range. These scores were further calibrated using an external sentiment lexicon (term: score) when lexicon terms appeared in the text:

$$S_{\left\{adj\right\}}=S_{\left\{orig\right\}}+(S_{\left\{dict\right\}}-S_{\left\{orig\right\}})\times \lambda$$

where $S_{\left\{dict\right\}}$ is the lexicon score and λ = 0.2. The result values were clipped to [−1, 1]. Lexicon matching was performed on the original full text to preserve contextual completeness, with calibration applied at the text level. For texts split into multiple segments, segment-level scores were averaged to produce a single text-level sentiment score. We then discretized sentiment into three categories using fixed thresholds: positive (>0.3), negative (<−0.2), and neutral (otherwise).

To validate the automated labeling, two coders independently annotated a reproducible random sample (random_state = 42), and inter-rater reliability was assessed using Cohen's κ (73). The coefficient between two manual coders was 0.752. The reliability metrics between the RoBERTa model and two human coders were 0.653 and 0.641, respectively, confirming substantial validity.

Finally, an engagement metric was operationalized by calculating a composite engagement score: (Shares * 0.4) + (Comments * 0.3) + (Saves * 0.2) + (Likes * 0.1). This weighting follows a “hierarchy of engagement” framework, designed to reflect the cognitive and social effort required for each action (74, 75).

On Douban, Shares are explicitly defined as sharing a broadcast so that followers can see it, representing the highest level of social effort. Shares are also the rarest in the dataset, with approximately 68% of posts receiving zero shares, making them a high-threshold indicator of endorsement. Comments represent substantial cognitive and social effort through active contribution, requiring users to articulate a response and participate in community dialogue. Within Douban's text-centric ecosystem, comments are the primary driver of thread longevity and community building, making them more significant than Saves and Likes. The high coverage of comments (only 6% of posts received zero comments) further reinforces their utility as a core metric of engagement. Saves indicate cognitive effort related to the perceived long-term utility of content, suggesting that users have processed the information and intend to revisit it, moving it to a personal resource. Likes are weighted lowest, as they represent a low-friction metric that requires minimal engagement or cognitive processing.

To test the robustness to the chosen weighting scheme, we recomputed engagement scores using (a) equal weights (0.25 each) and PCA-derived^3^ weights. The Spearman rank correlation between this study's composite engagement score and the alternative model was exceptionally high ($r_{s}$ > 0.99), confirming that this study's engagement metric reflects the underlying structure of engagement in the data, regardless of the specific weighting scheme.

All computational layers were interpreted inductively and grounded in FNM and intersectionality, ensuring an empirically grounded yet theoretically attuned analysis. Rigor was established through the triangulation of computational metrics with qualitative observation. On OSF, we provide separate files for (a) field notes; (b) anonymized engagement-sorted posts and comments; (c) the stopword list; (d) cleaned and segmented text data; (e) the inter-topic distance map; (f) RoBERTa-based sentiment analysis; (g) the Seed 42 validation sample (*N* = 500); and (h) manual coding datasets for validation from two coders; and (i) the top 1% quotes by sentiment.

## Results and discussion

### Becoming-infrastructure: participatory governance and feminist knowledge-making

In this paper, relationality refers to how members' bodies, emotions, and knowledge claims become meaningful through interactions with others and with community-mediated features, including posts, comments, likes, and moderation actions like pinning, highlighting, deletion, and entry screening. We try to trace how these relational practices are produced, stabilized, and contested in engagement and how they become sedimented within the community infrastructure.

When *Women Fitness* launched in July 2022, it lacked formal community guidelines. Several early posts focused on aesthetic ideals, such as “slimming thick arms” and the “perfect waist-to-hip ratio” (Table 1). However, these were immediately contested by members who mobilized feminist and fitness literacies to foreground body autonomy and plurality. By challenging pseudoscientific misconceptions, *Women Fitness* actively rejected unattainable and unhealthy body ideals (Table 2).

These grassroots practices were later supported by administrators through entry screening, pinned “must-read” archives, and moderation rules (Table 3); introduced in September 2022, these rules banned pseudoscience, male-gaze framing, body ideals, advertisements, identifiable photos, and celebrity-related content (https://docs.qq.com/doc/DZHVuTG1PYmJTSEZt) and were enforced through content deletion and membership stripping. In this sense, governance within *Women Fitness* did not precede participation but emerged organically from it, a process this paper terms becoming-infrastructure.

By restricting advertisements, identifiable photos, and celebrity-related content, *Women Fitness* counters neoliberal self-branding and disrupts algorithmic virality, thereby challenging dynamics typical of mainstream fitness culture. These rules actively resist neoliberal constructions of the “self-as-project” (4, 76, 77) and the monetization of low fitness literacy and the algorithmic amplification of clickbait-driven misinformation (78, 79). Instead, the community infrastructure promotes critical inquiry over influencer culture, reinforcing a shared commitment to feminist and fitness literacy, thereby enacting what Braidotti (80) describes as a “threshold of sustainability”—an affirmative ethics that seeks to increase the capacity of a body or a community for action while resisting the self-destructive exhaustion inherent in neoliberal performance.

Computational analyses validate this digital ethnography. Topic modeling confirms that the most prominent thematic clusters prioritize sustainable training knowledge, such as movement mechanics, recovery strategies, injury prevention, and adaptations related to menstrual cycles and equipment accessibility (Table 5), highlighting how members adapt to material constraints by recommending home-based workouts, free or low-cost gear, and free training resources. These adaptations reveal members' efforts to produce knowledge that accounts for sociocultural inequality. Engagement metrics further demonstrate that content resisting body ideals and consumerist narratives receives high interaction (Tables 6, 7).

**Table 5:** Thematic overview.

Topic	Keywords	Posts	Proportion (%)	Summary
Muscle training over weight loss	Body fat percentage, home-based, women, meal, height/weight, clothing, mood, breaststroke, diet, my whole body	1,230	26.97	Embracing strength training over weight loss, focusing on muscle gain, home workouts, and emotional well-being
Fitness struggles	Ribs, calf, home-based, inner side, front side, speed, hyperextension, back, stiff, message	726	15.92	Common body postural and muscle issues encountered during workouts
Accessibility	Studio, detach, membership, home-based, lean forward, one session, training log, group class, boxing gloves, free	700	15.35	Choices around accessing fitness spaces and gears
Nutrition, rest, and emotion	Eat, protein, calf, home-based, body fat percentage, rehabilitation, calories, fatigue, not eat, mood	699	15.33	Interplay between diet, emotional well-being, and fitness performance
Comfort and injury prevention	Shoes, sneakers, running shoes, calf, drag, front side, lateral side, socks, scapula, curl	502	11.01	Training form and gear alternatives
Daily fitness emotions and challenges	Power clean, praise, embarrassing, super happy, bottled water, report, typo, this, service fee, pole position	324	7.10	Emotional fluctuations
Exercise-induced conditions	Bleeding, cold, symptom, resting, menstruation, cough, dizziness, ovulation period, heart, headache	188	4.12	Symptoms and medical concerns
Nutrition strategies and struggles	Protein, chicken breast, dinner, egg, bread, rice, meal, lunch, breakfast, three meals	182	3.99	Balancing healthy eating with life constraints

**Table 6:** Top 10 popular posts.

No.	Title	Comments	Likes	Saves	Shares	ES^a^
1	To prevent fitness beginners from taking unnecessary detours, will share small fitness tips that we know	186	4,658	13,824	1,891	4,042.8
2	Swimming plus other exercises is the ultimate combination	139	4,241	3,022	531	1,282.6
3	Push-ups from scratch	82	1,386	4,553	502	1,274.6
4	A simple explanation of the principles and methods of strength training	158	1,739	3,403	347	1,040.7
5	Guidance for beginners♡ important community updates	130	655	3,885	386	1,035.9
6	Leg training for 4 months	64	1,332	2,966	299	865.2
7	Changes after losing weight and gaining muscle	98	1,028	2,725	278	788.4
8	Shoulder and back training	42	1,095	2,738	254	771.3
9	Effective low-BMI home workouts	36	732	2,783	235	734.6
10	For those sisters who have cold hands and feet, sharing autumn/winter exercise tips	51	1,031	2,195	343	694.6
…	…	…				

a It refers to “engagement score.”

**Table 7:** Top 10 comments.

No.	Comment	Likes
1	My nipples showing only proves that I have nipples	7,263
2	Men's bulge in tight clothes is what's actually embarrassing. What's there for women to be embarrassed about? They just want to try to make us feel ashamed and then make a fortune out of it	6,517
3	Swimming clears my mind. It truly feels like being in a state of “flow”	4,813
4	Yes! If there were a women-only gym in my city, I'd definitely go. I don't go to gyms now because all gym members are mostly men. I can't stand the way they look at me	3,806
5	There's nothing embarrassing about being a woman	3,586
6	Yes, many women don't go to gyms because they don't want to be around men	3,283
7	I'm not hungry because I drank a lot of pool water	3,091
8	Don't let men know about this, they are going to flip	2,720
9	This reminds me of women's swimsuits are often with all kinds of frills. But professional swimmers would never wear those	2,681
10	We women should live as humans rather than pets. We need the ability to protect ourselves in times of need. The more we eat, the higher our chance of surviving. The more we train, the better our chance of fighting back	2,561
…	…	…

Furthermore, members critique mainstream sport science while simultaneously mobilizing science as a protective boundary. On the one hand, community discourses challenge male-default fitness knowledge that has overlooked women's hormonal cycles, biomechanics, and recovery patterns (81–84). For instance, members rejected mainstream fitness practices such as fasted morning cardio, noting that such approaches are often optimized for male metabolisms and can trigger hormonal imbalances or endocrine disruption in women. Members also reframe “progress” away from linear increases in weight or intensity toward menstrual periodization, acknowledging that recovery and strength are non-linear and tied to cyclic physiological shifts. Threads like “Train with a Women-Centered Mindset” and “Rugby for Women-Centered Beginners: A Guide” (see fieldnotes on OSF) thus rework scientific knowledge through women's lived specificities, including tendonitis, aging, food-related anxieties, and insomnia, in ways that affirm sustainability, body plurality, anti-ableist commitments, and sensitivity to diverse socio-economic constraints (Table 4).

Together, these patterns indicate that feminist epistemic commitments are not only articulated but actively rewarded within *Women Fitness*. In other words, the community's infrastructure systematically incentivizes feminist knowledge production. At the same time, this women-centered reworking does not entail a complete rejection of scientific authority. Rather, members frequently invoke biomedical legitimacy to differentiate “evidence-based” guidance from fitness misinformation and patriarchal pseudoscience. In practice, feminist epistemic commitments are rewarded not only when they can be articulated in a recognizable “scientific” register, through physiological explanations, safety-oriented risk framing, and academic research. However, this evidentiary style is unevenly accessible, as not all members have the resource to evaluate the reliability of those results. The result is a paradox in which science becomes both the object of critique and a tool of community governance. While such boundary work helps protect members from harmful misinformation and beauty standards designed to shrink women, it can also render scientific competence an implicit prerequisite for full participation. As moderation practices become formalized, these well-intentioned epistemic literacies may become unspoken gatekeeping norms that disproportionately disadvantage women from marginalized class or educational backgrounds, echoing intersectional critiques of universalizing feminist claims.

Thus, *Women Fitness* exemplifies the promise and complexity of becoming-infrastructure—a dynamic, reflexive process shaped by internal power dynamics, unequal access, and ongoing struggles over what counts as legitimate knowledge.

### Becoming-affect: emotional labor and communal force

**Figure 1:**
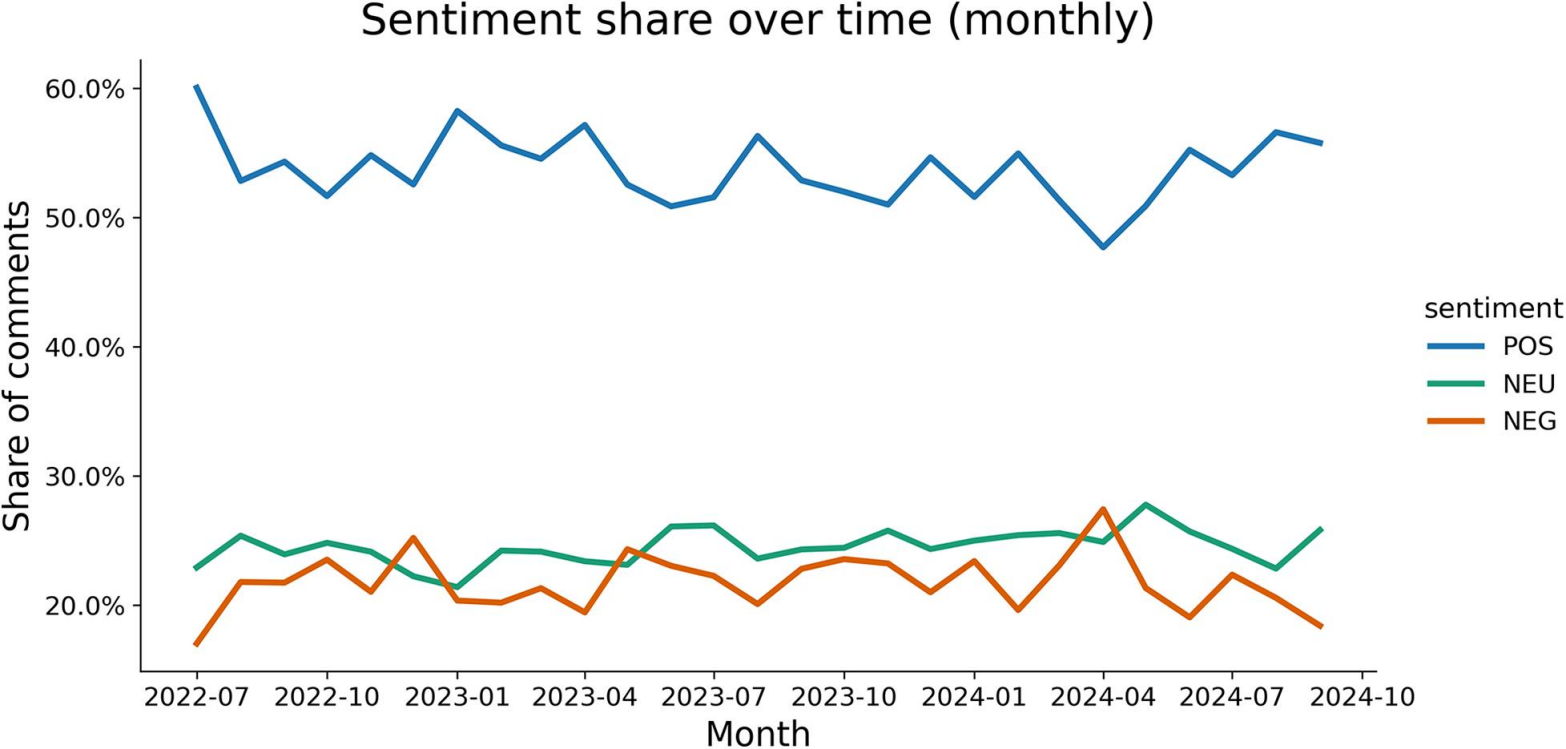
Sentiment over time.

The affective landscape of *Women Fitness* is characterized by a sustained orientation toward affirmative becoming. Across the longitudinal distribution of sentiment (Figure 1), positive affect remains the dominant register, while neutral and negative sentiment fluctuate within a lower band.

**Figure 2:**
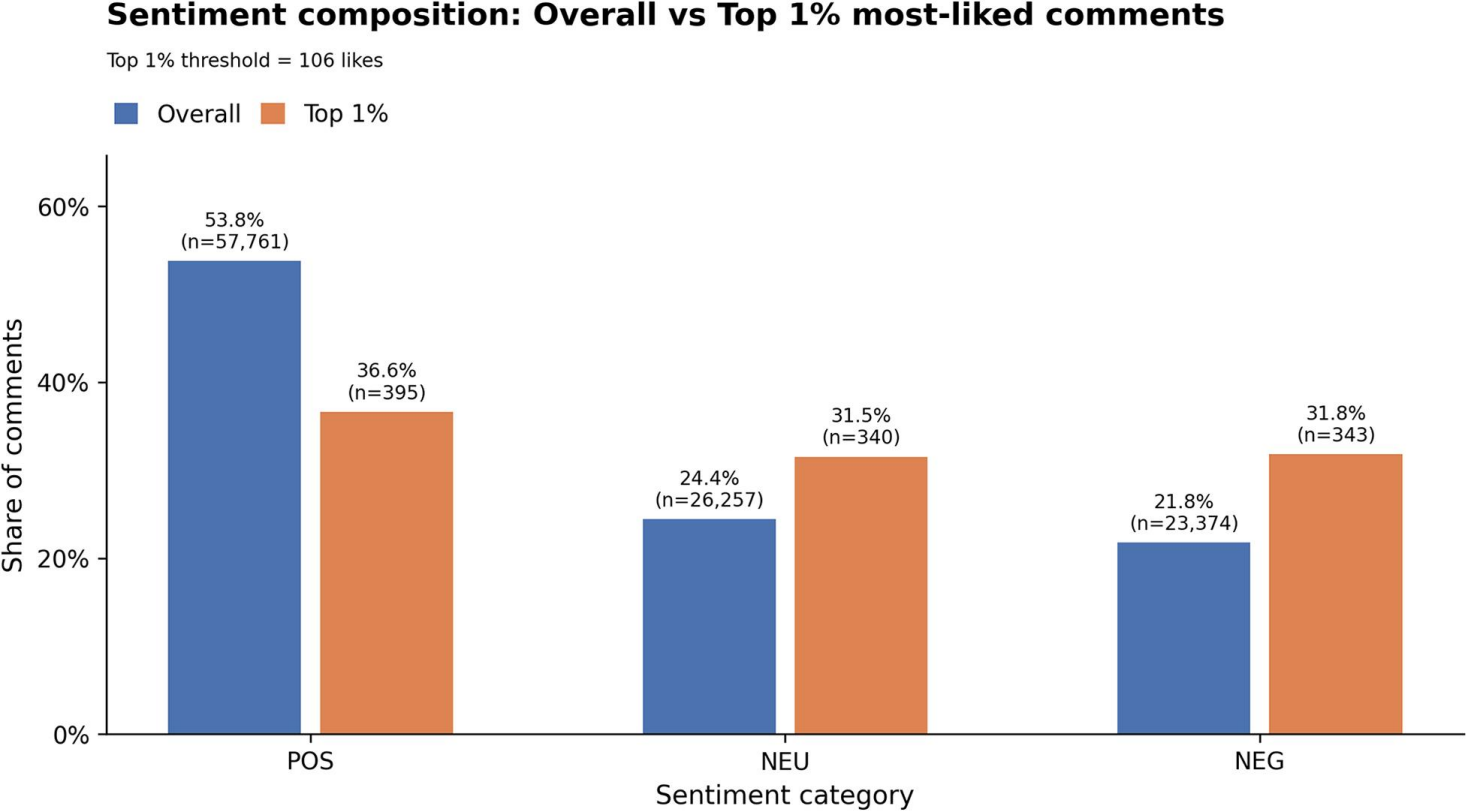
Sentiment composition.

The aggregate sentiment composition (Figure 2) reveals that 53.8% of comments are positive, 24.4% are neutral, and 21.8% are negative. This contrasts with the toxic affective climate of Thinspo and Fitspo cultures, where it intensifies self-surveillance and body dissatisfaction (23, 24, 26). Among the top 1% of most engaged affects, the distribution becomes more even (36.6% positive, 31.5% neutral, 31.8% negative), suggesting that visibility is not monopolized by positivity but is allocated across affects that travel through the community's engagement.

Table 8 clarifies what specifically becomes amplified. The most-engaged affect is neutral (“My nipples showing only proves I have nipples”), indicating that *Women Fitness* strongly circulates low-friction, repeatable formulations that prioritize body autonomy and deterritorialize shame. Neutral affect thus operates as a carrier of shared feminist literacy that circulates effectively, allowing autonomy to be enacted through ordinary speech acts (85). At the same time, it amplifies negative affect when articulated as critique and collective resistance. High-engagement negative comments condemn gendered design, commodified femininity, historical misogyny, and the male gaze, while preaching urgent safety knowledge. In these cases, negative affect becomes circulatory and actionable through relational uptake, including agreement, repetition, humor, and practical advice, rather than being dismissed and privatized (57, 85, 86). Neutral affect often acts as a low-friction carrier of feminist common sense and everyday tactics. Statements such as “My nipples showing only proves I have nipples” and “Jump rope… keep a one-meter distance” help stabilize a shared orientation toward bodily autonomy and pragmatic agency. Positive affect, finally, consolidates these forms of resistance and transforms them into affirmative orientations. For instance, mutual recognition (“Fat and muscle are both girls' best friends”) and strength-as-survivability (“live like a human rather than a pet”) frame fitness as a means of expanding the margins of health and safety.

**Table 8:** Most engaged affect by sentiment.

Sentiment	Likes	Post title	Comment quote
NEG	6,517	What a stupid design	Men's bulge in tight clothes is what's actually embarrassing. What's there for women to be embarrassed about? They just want to try to make us feel ashamed and then make a fortune out of it
NEG	3,806	Do you think women-only gyms have a market?	Yes! If there were a women-only gym in my city, I'd definitely go. I don't go to gyms now because all gym members are mostly men. I can't stand the way they look at me
NEG	2,571	Women are not physically weak	Women are weaker, only because that's the result of over 3,000 years of being brainwashed into shrinking and belittling ourselves. In matriarchal societies women were taller and sturdier, we were very strong. Even today, women's lower bodies are still as strong as men's. Build upper-body strength, eat more meat/eggs/dairy, starting from our generation we can change this “omen are physically weak” situation
NEG	1,670	Cardio + a massage gun caused my ovarian cyst to rupture	Don't massage-gun the belly!! Don't do joints!! Don't do bones!!
NEG	1,345	Why is fitness gear marketed to women so expensive?	Pink tax
NEU	7,263	Why wear padding when swimming? Doesn't braless feel awesome?	My nipples showing only proves I have nipples
NEU	2,491	Girls, any exercises you can do while waiting in line for PCR test?	Jump rope, it also automatically makes the people in front/behind you keep a one-meter distance
NEU	1,764	Never encouraging others to work out again	I fully support men not going out working, as long as they pay full social insurance and housing fund
NEU	1,534	Cried in a group class today	Sis, don't blame yourself! You're the customer, you're the boss. If you couldn't learn it, that's on their incompetence! […] Introverted girls should remember: reflect on yourself less, blame others more!
NEU	1,428	Is this a muscle that normal people have?	You can go look around and do some research at the gyms. Most beginner men don't have arms like that. Men are very used to belittling women, that's all
POS	2,561	Few thoughts on the South Korea crowd-crush tragedy	Only when women live like humans rather than pets can we protect ourselves when a crisis hits. The more we eat, the higher our chance of surviving. The more we train, the better our chance of fighting back
POS	1,669	Wished a stranger “Happy Women's Day” at the gym, but she said she doesn't like Women's Day	It's okay, I worked out today and I'll say it to you: “Sis, happy Women's Day!”
POS	1,055	Why does my belly look both skinny and fat…?	Sis, don't insult your body like that. It's good. Fat and muscle are both girls' best friends. If you like a body with a thin layer of fat, keep doing cardio then
POS	1,038	Bouldering cured my body anxiety	So badass! your shoulders and back look powerful and really beautiful
POS	1,030	Share workout tips you know to help beginners avoid detours	Girls' “main focus” isn't just glutes. For beginners, shoulder and back gains show up really quick! Use the cable machine and small dumbbells, gradually add weight, and you'll see results quickly

Taken together, within *Women Fitness*, vulnerability and fluctuation are not treated as failure but as legitimate states that invite care, recognition, and feminist critique. This pattern resonates with FNM's conceptualization of affect as a transindividual, emergent force (85–87) and with “affirmative ethics,” in which negative affect is not denied but transcended into collective labor for creating active possible futures. In this way, vulnerability is transformed into feminist resistance, illustrating how affect circulates as a force of political and ethical reorientation (88).

Community moderation further fuels this affective economy. By banning terms such as “A4 waist” and “mom hips,” *Women Fitness* redirects toxic affect outward while fostering internal solidarity. Meanwhile, formerly stigmatized expressions such as “猛女” (fierce woman), “大女人” (big woman), “太娘们了” (very womanly), and “蒂蒂” (clity) are reterritorialized as affective anchors of joy, pride, and defiance. As affect is redistributed and intensified, the community reorganizes its affective economy, expanding its collective capacities for resistance, care, and becoming (85, 88). These affective exchanges constitute intra-active reconfigurations, in which emotions are not private but material-discursive, where the interface features, such as likes, comments, highlighted and pinned threads, act as conduits for the circulation of intensities (15, 85). In this way, negative affect is metabolized into what Braidotti (60, 88) calls affirmative becoming: the capacity to move through pain without becoming fixed by it.

However, this affective labor is often unevenly distributed. Some members may hesitate to share or be triggered by such content due to unfamiliarity with feminist discourse or a lack of emotional resources. As emotions circulate differently based on positionality (89), *Women Fitness*, despite its supportive aims, reflects and reproduces structural inequalities in affective labor.

### Becoming-Bodies: decentering the ideal body

The body is reconceptualized as a site of lived struggle and feminist potential. This aligns with Deleuze and Guattari's (90) concept of “becoming-body,” which posits embodiment as relational, fluctuating, and non-linear, as well as with FNM accounts of the body as an active, agentic, and environmentally embedded assemblage (58, 88, 91). Within the community, fitness is organized around fatigue, aging, menstrual cycles, injury prevention, nutrition, and emotional and physical fluctuations (Tables 4, 5, 8). Topic modeling and engagement metrics confirm the centrality of this knowledge-sharing ethos, in sharp contrast to the aspirational fitness discourses typical of mainstream fitness cultures.

Members explicitly reject the very necessity of body ideals. As one member writes, “The ‘ideal' body is just a patriarchal fantasy, which women have every right to reject and ignore” (Table 2). Another reframes fitness in terms of autonomy: “The more we eat, the higher our chance of surviving. The more we train, the better our chance of fighting back” (Tables 7, 8). Such statements exemplify what FNM describes as a shift from representational bodies to lived, material, and affective forms of embodiments (15, 88).

Community guidelines formalize moderation practices that support this post-representational politics of embodiment. By banning visual comparisons and maintaining pinned threads such as “Say Goodbye to Body Image Anxiety” and “Train with a Women-Centered Mindset” (see fieldnotes on OSF), *Women Fitness* actively fosters empathetic, care-based participation. Through these practices, non-normative bodies are validated and the scope of “fitness” is expanded to include diverse bodily capacities, histories, and needs, directly addressing Rice et al.'s (44) critique of the ableist and exclusionary nature of Body Functionality.

This orientation reflects an FNM understanding of the body as always in relation to affect, technologies, environments, and social structures (15, 58, 91). Within *Women Fitness*, the body is allowed to be strong, tired, injured, aging, menstruating, or at rest. Such decentering enables the embodiment of what Braidotti (88) terms embodied multiplicity, in which bodies are, but to become, in care, in movement, and in resistance.

At the same time, the feminist reconfiguration of the body remains materially constrained. Unequal access to rest, recovery, healthcare, or safe training continues to limit body autonomy in practice. Moreover, although fitness is reframed as a form of agency and self-defense, this framing—while pragmatic—can still reproduce patriarchal logics that equate strength with safety (Tables 7, 8), as well as neoliberal logics of individual self-responsibilization (4, 76). While *Women Fitness* effectively dismantles internalized burdens such as body image anxiety and productivity guilt, structural violence remains an external boundary that the community cannot fully transcend. As a result, its embodied practices remain entangled with patriarchal and neoliberal pressures it seeks to resist.

### Becoming-subjects: feminist alignment and performed belonging

In *Women Fitness*, subjectivities emerge as the cumulative yet contested effect of governance, affect, and embodiment. The community functions as a material-discursive apparatus (15), in which the body, through pain, fatigue, and strength, becomes meaningful through its intra-actions with collective infrastructures, norms, and affect. Instead of pre-existing this environment, subjects are continually materialized through repeated practices of resistance, care, refusal, and adaptation—an onto-epistemological process in which knowing, being, and doing co-emerge (14, 15).

These processes constitute what Braidotti (60, 88) terms posthuman subjectivity—a relational, embodied, and affective mode of being that is not grounded in liberal individualism but in shared capacities for becoming. Feminist alignment within *Women Fitness* is thus enacted through material practices of embodied training, affective exchange, and moderation, which shape members as gendered, athletic, and politically situated subjects (58, 85).

Crucially, this alignment is an interaction accomplishment rather than a given. Relationality is actively produced, recognized, and stabilized through recurring discussions that construct a collective “we,” using terms such as “sisters (姐妹)” and “fellow fierce women (各位猛女)”; affirmation through the deterritorialization and reterritorialization of shame and value (“don't insult your body” and “muscles are girls' best friends”); and the provision of women-centered, safety-conscious practical guidance. These practices function as relational care that translates embodied vulnerability into shared belonging and actionable knowledge.

Yet this process of becoming-subjects is always shaped by structural inequalities. Belonging within *Women Fitness* requires epistemic literacy, affective capacities, and, at times, bodily resources, all of which are unequally distributed across axes of privilege (17, 18). This highlights the paradox of feminist counterpublics: while *Women Fitness* opens new possibilities for feminist becoming, it can also reproduce exclusions in epistemic, affective, or embodied terms, which inadvertently privilege those most able to perform feminist competence, emotional labor, or bodily resilience (62, 89).

Nevertheless, *Women Fitness* materializes Braidotti's (88) concept of the “politics of location” in posthuman form, a situated, collective, and materially grounded feminism that does not promise universal inclusion but instead offers contingent yet powerful modes of resistance and care. The feminist subjectivities produced here are neither autonomous individuals nor passive victims but relational agents whose capacities are continually negotiated in tension with the patriarchal and neoliberal structures they cannot fully escape. Through its infrastructures of knowledge production, affective circulation, and embodied practice, *Women Fitness* provides a practical blueprint for how posthuman feminist subjectivities can be assembled within contemporary digital fitness cultures.

## Limitations and future directions

This study is limited by its focus on a specific community on Douban, a platform defined by knowledge-centered affordances that tend to cater to a demographic with high levels of cultural capital and consumption. Consequently, the findings are subject to sampling bias: Douban users are not representative of the broad population of Chinese women who engage in fitness, nor do they reflect the full spectrum of socio-economic, regional, and linguistic diversity across the country. Future studies should therefore explore cross-platform and cross-cultural comparisons.

Moreover, the computational analysis prioritized textual data, thereby excluding visual and multimodal elements such as photos, videos, memes, and emojis. Multimodal methods are needed to account for image- and video-based content, providing a more layered understanding of online expression.

Finally, it is also important to acknowledge the inherent limitation of digital ethnography: the inability to directly infer offline behavior from online engagements. While the findings capture engagement patterns and digital subjectivities, they do not necessarily equate to members' lived experiences in real life. Further research incorporating qualitative interviews is needed to bridge the gap between online and offline practices.

## Conclusion

This study demonstrates how feminist digital communities can reconfigure fitness from an individualized, aestheticized, and monetized project into a collective practice of becoming, care, resistance, and knowledge production. Through an integrated analysis combining digital ethnography, topic modeling, sentiment analysis, and engagement metrics, this paper shows how *Women Fitness* systematically redirects attention away from body ideals, influencer impacts, and neoliberal self-optimization toward a sustainable, relational, and pluralistic practice grounded in feminist solidarity.

The community's becoming-infrastructure reveals that governance is not a prerequisite for participation but an organic outgrowth of it. By formalizing moderation practices that support resistance, *Women Fitness* enacts Braidotti's (80) concept of “threshold of sustainability.” Becoming-affect is central to *Women Fitness*'s transformative power; it facilitates the metabolism of negative affect, transcending and transmuting isolated negative experiences into shared forces of belonging and potential. Similarly, becoming-bodies are reframed through the vibrant materiality of aging, menstruation, injury, fatigue, and recovery, allowing for an embodied multiplicity that affirms bodily plurality and disrupts normative narratives of progress.

Based on these findings, this paper offers the following evidence-based recommendations for practitioners, digital moderations, and feminist collectives. (1) Participatory governance: the digital ethnography suggests that administrators should adopt a reflexive approach in which community guidelines are not predetermined or fixed but instead emerge from members' engagement and contestation, thereby supporting such grassroots resistance to protect the community's epistemic boundaries. (2) Affective labor and peer support: the sentiment metrics and the transformation of negative affect found in the findings suggest that collectives should center care-based emotional labor that validates negative affect and actively reworks it into shared literacy and collective agency. (3) Materialize accessibility: to avoid the epistemic exclusions identified in this study, collectives must balance their commitment to scientific legitimacy with an understandable, entry-level way of wording. Efforts must be made to ensure that fitness and feminist literacy do not reproduce an unspoken prerequisite that excludes those with fewer privileges. Collectives should actively decenter the body by centering sustainability and accessibility. This involves sharing situated knowledge on free or low-cost resources to account for the material inequalities of the “politics of location.”

Ultimately, *Women Fitness* exemplifies the emergence of posthuman feminist becoming-subjectivities—relational agents whose capacities are continually negotiated in tension with the neoliberal and patriarchal structures they aim to dismantle. While *Women Fitness* cannot fully transcend structural violence, it provides a practical blueprint for feminist resistance within digital fitness cultures. Feminist fitness, then, involves embracing the ongoing, situated process of staying with the trouble, recognizing contradictions as the very conditions of becoming.

## Data Availability

The datasets presented in this study can be found in online repositories. The names of the repository/repositories and accession number(s) can be found below: https://osf.io/re8vj/overview?view_only=8ac2d6a73f4145d0be2f0038257e6724.

## Footnotes

^1^Abbreviation for “bonespiration,” a portmanteau of “bone” and “inspiration.”

^2^Abbreviation for "Robustly Optimized BERT Pretraining Approach."

^3^Shares (0.08), Comments (0.17), Saves (0.468), Likes (0.279).
