# Supplementary material for: Becomings beyond the ideal: women's fitness and digital subjectivities in the Douban women's fitness community
Source: Front Sports Act Living. 2026 May 1;8:1786979. doi: 10.3389/fspor.2026.1786979 (PMC13176220; doi:10.3389/fspor.2026.1786979)
Supplement: Supplementary file 1 [file Supplementaryfile1.docx]

Community Guidelines

- 1. Daily Reporting and Community Affairs Dedicated Thread

This thread is for reporting only. Please do not reply unless you are submitting a report.

1. Zero Tolerance for Male Accounts

- No men allowed in Women Fitness. Men who somehow manage to trick their way into our community will never get hard ever again.
- If any male users are found lurking or harassing members in any way, they will be permanently banned.
- Do not post images that contain unblurred male figures.

1. No Personal Attacks or Exercise Shaming

- Respect all forms of exercise, there’s no such thing as “better” or “worse” workout. Whether it’s cardio, strength training, or yoga, all are valid personal choices.
- Do not criticize or belittle others’ fitness preferences or progress.

1. No Advertising, Socializing, or Non-fitness Content

- No advertisements, friend-seeking, group invitations.
- Celebrity-related and any vulgar or sexual content are strictly prohibited.

1. No Repeated Check-in Threads

- Each member may only create one check-in thread.
- Please update within that thread instead of starting multiple threads.

1. No Male-Pandering Content

- Any content that seeks male validation or reflects internalized male-gaze is not allowed. (Women Fitness Administrator, 2022b)
  1. About recent discussion about the obsession of low body fat

Reminder: Women Fitness is a community focused on women’s fitness, not on weight loss or improving appearance. If you are concerned with these issues, please find another community that suits you.

Recently, certain posts have caused controversy. We have received member complaints about content promoting an obsessive focus on low body fat, the pursuit of genetically unattainable body standards, and body anxiety rooted in the male gaze.

1. Core Mission: The purpose of Women Fitness remains to be a female-only fitness community that advocates for “Health, Confidence, and Happiness.” Health comes first. Choose a workout method that is suitable for your own condition. Some members have mentioned that this community is becoming a “weight loss community”. For those starting with a higher body weight, there is nothing wrong with losing weight for health reasons. The key is the motivation. We hope members can be more inclusive and supportive of everyone who is slowly improving themselves for the sake of their health.
2. Reject Body Anxiety and the Male Gaze: Excessively pursuing low body fat, a specific waist-to-hip ratio, an inverted triangle physique, etc., may be a personal choice. However, this choice is often a “downward freedom” that has been disciplined by the male gaze. In today’s society, while it’s difficult to completely escape this aesthetic disciplining. For example, aren’t bodybuilders also seeking broad shoulders and shapely glutes for the visual effect of a smaller waist? The key difference lies in how one pursues it. Some choose to fight for more space and freedom (e.g., by choosing to build significant muscle), while others choose shortcuts (e.g., extreme dieting to control measurements).

Please try to make peace with your body. Body shapes are natural, whether it’s pear, apple, or hourglass, each type has its own pros and cons. It is far more meaningful to work with your natural characteristics to shape your body than it is to despise it. In doing so, your fitness journey will gradually help you transform the male-gaze aesthetic. It won’t just be about “beauty”; you will also become more confident and powerful.

1. Understand Basic Fitness Knowledge. This includes, but is not limited to: “Focus on body fat percentage, not body weight” “There is no such thing as spot reduction (localized fat loss)” “Genetics are innate, fitness cannot change your fundamental body type (e.g., it can’t give you slender ankles if you don’t have them naturally), and “Adjust your diet structure, don’t just go on an extreme diet.”

The group will be adding a “Fitness Science/FAQ” section to categorize basic fitness knowledge. Before asking a question, please use the group search function to avoid repetitive, low-effort posts. Threads asking questions about basic fitness facts that have already been covered in the FAQ will be locked.

1. [NEW] Addressing Dissatisfaction with a Specific Body Part: If you are unhappy with a certain part of your body, first consider if it can be changed through muscle gain or overall fat loss. Remember, there is no such thing as localized fat loss. If you don’t know how to train for it, you can ask for advice in the group. However, if it's something that fitness cannot change, please do not post about it. There is no need to be overly critical of every little bit of flesh on your body. Focus on building your larger muscle groups and developing a solid overall frame first.
2. [NEW] Respect Individual Differences and Seek Professional Help: While human body structures are broadly similar, details like bone frame size are determined by genetics. This group is for rational discussion about fitness. We are not fortune-tellers or doctors. If you experience any physical discomfort, please go to a proper hospital. Seeking help from professionals is always better than asking netizens. For non-pathological issues, please respect your genetics. For pathological issues, please go to the hospital and seek for professional help.

To more clearly illustrate the types of posts that are prohibited under the community guidelines, the following examples are known to result in the threads being locked and warnings issued.

1. Inquiries about how to reduce or address a lower belly pooch, protruding abdomen, or anterior/ posterior pelvic tilt.
2. Questions regarding how to slim specific body parts such as calves, thighs, the gastrocnemius or the triceps.
3. Posts focused on perceived “unflattering lines” on particular parts of the body.
4. Concerns about localized indentations or bulges of fat or muscle.
5. Posts related to the idea of “serving beauty”, including “outfit of the day” “looks good” “pretty” or other appearance-focused comments.
6. Complains about a lack of visible physical change after a short workout period (e.g., less than three months).

Members who encounter posts violating these guidelines are encouraged to report them in the Daily Reposting and Community Affairs Thread.

Finally, Women Fitness encourages all members to learn actively, observe attentively, and practice consistently. Members are advised not to rely solely on free advice and are discouraged from engaging in performative participation. Verbal engagement alone does not equate to genuine understanding or action. (Women Fitness Administrator, 2022a).

Helpful Tips and Guides for workouts

1. For fat loss, it’s better to do anaerobic before aerobic. First, use anaerobic exercise to quickly deplete your glycogen stores for approximately 45 minutes, then use aerobic exercise for sustained fat burning for 30 minutes to 1 hour.
2. Eating fast-digesting carbs after a workout can quickly help your body enter an anabolic state. Fast carbs include things like steamed buns, rice, noodles, watermelon, bananas, etc. You can look them up yourself. (Adjust based on your personal situation. For example, if you finish your workout too late at night, you might skip this).
3. Diet during a fat-loss phase is extremely important! (I personally think it’s more important than training). The cooking method is key. For example, if you stir-fry vegetables in a large amount of oil, how many vitamins will be left?
4. You really don’t need to specifically train small muscle groups like abs. They will become visible once your body fat percentage drops. Focus on training large muscle groups! Like glutes, legs, and back.
5. It’s very normal for your body to retain water (edema) due to water and sodium retention before and during your period, which will cause your weight to go up.

--- Updates Compiled from the Comment Section ---

1. When climbing stairs, focus on using your glutes to power the movement. In your daily life, engage your core more, whether you’re walking, climbing stairs, or sitting at your desk. Keep your abs tight.
2. Keep your workouts varied. This helps for the sake of enjoyment and also for balanced development.
3. Losing weight on the scale doesn't equal fat loss, and exercising doesn’t guarantee the number on the scale will drop. Pay more attention to changes in your body measurements. Exercise moderately, rest when needed, and focus on your sleep schedule. Good sleep is essential for fitness! Poor sleep, especially during fat loss, affects growth hormone and can prevent you from losing fat.
4. During fat loss, be sure to supplement with healthy fats (like nuts) and drink more water! You must drink enough. The water from fat metabolism is expelled through urine, and the carbon dioxide is expelled through breathing.
5. Unless you have a high body weight or the “three highs” (high blood pressure, high blood sugar, high cholesterol), there is no need to do a ketogenic diet. The keto diet is truly not healthy. Refined grains like rice and flour are carbs, just like purple sweet potatoes and corn. You don't need to fear rice and flour. During a fat loss phase, you can replace them with whole grains as your staple food. The reasons are that their calorie density is different and low-GI foods don't cause major insulin spikes.
6. You can only build muscle in specific areas; you cannot spot-reduce fat! For example, there’s no such thing as only slimming your legs, only slimming your waist, or only slimming your belly. Fat loss happens across the entire body, it’s just that the speed and order differ for each person. Fitness also won’t change your genetics, like giving you slender ankles or widening a narrow hip structure.
7. Whether you’re building muscle or losing fat, don’t set your goals too big. Accomplish small goals one stage at a time.
8. Treat fitness as a habit. Stick with it for at least 3 months before you start looking for changes in your body. Don’t be impatient for quick results.
9. If you are strictly controlling your diet, it’s very necessary to schedule a cheat meal every once in a while.
10. Don’t get discouraged by a plateau. Maintain your habits, and your weight will continue to drop after the plateau period.
11. Fat loss isn’t just about doing cardio, is about strength training also. Doing only cardio (especially without enough protein) can lead to muscle loss, turning you into a “skinny-fat” person. The benefit of strength training is that the results are less likely to rebound.
12. Training your shoulders and back well will make your entire posture more upright and give you more energy.
13. The idea that “light weight and high reps” can “tone” or “sculpt” your body is a misconception. This likely means that light weights don’t stimulate the muscle enough. If you don’t feel anything the next day, it was probably an ineffective workout.
14. Coordinate your breathing with your exercises. Don’t hold your breath. Many experienced members in the group talk about the “mind-muscle connection” during strength training, and it’s true. Also, remember to relax when you stretch.
15. During your ovulation period, you may need to control the intensity and number of sets in your workout, otherwise, it can easily lead to spotting (breakthrough bleeding).
16. For beginners who don’t usually exercise, it’s very normal for your weight to increase when you first start. This is because your muscles are storing water. Stick with it and don’t get anxious staring at the number on the scale.
17. It’s natural for women to have a small lower belly pouch because we have a uterus and ovaries. It’s completely normal for your belly to stick out after sitting or eating.
18. During strength training, focus on the mind-muscle connection, think about the muscle you’re training. You can start with a light weight such as 5kg to find the feeling of that muscle group activating. Once you’re used to it, slowly increase the weight. For heavy weights, follow the breathing pattern of “inhale, hold breath, exhale” during the exertion.
19. Progressive Overload Example: After warming up, do your first set with your usual working weight. For the second set, add 5kg, but do 2-4 fewer reps than usual. Then repeat this cycle.
20. For strength training, depending on your situation, you can arrange 4-6 sets for each exercise, with 10-15 reps per set. A workout for one body part should include about four or five different exercises. Note: After a while, you might stop feeling it. That’s a sign to either change the exercise or increase the weight.
21. Your training plan should be adjusted based on your weekly frequency, whether it’s a full-body routine, a two-day split, or a three-day split. If you train four times a week, you can choose a two-day split (e.g., upper body/lower body). For six days a week, a three-day split focusing on large muscle groups is recommended. If you can only train for two days, a full-body routine is likely the better choice.
22. I recommend a great fitness app called “训记”(Xùn Jì). How to use it: When you train, tap the “+” in the middle to create a new workout, add your exercises, and you can record your sets and weights. The next time you do the same exercise, it will automatically carry over the weight, which is very useful.
23. For running, please wear specialized running socks or toe socks to reduce friction between your toes and prevent blisters. To become a better runner, you need to strengthen your lower body muscles (squats, single-leg figure-eights, etc.) to prevent insufficient muscle strength from putting a burden on your knees.
24. Interval running burns more fat and is more beneficial for improving cardiopulmonary function than just running at a steady pace. Don't skip your warm-up before and your cool-down stretch after!
25. You must schedule rest days from running. In the beginning, you can try running one day and resting one or two. As you adapt, you can increase to run-two-rest-one, run-three-rest-one, etc. When running, focus on increasing your cadence (step frequency) and reducing your stride length (i.e., take smaller steps). Your foot should land directly under your body, not out in front of it.
26. Pay attention to stretching before and after exercise. Do dynamic stretching before your workout and static stretching after.
27. In strength training, focus on the eccentric! Pay attention to your tempo! A slow eccentric (lowering) phase is very important for muscle hypertrophy (growth).
28. You must prioritize sleep! Muscle repair after a workout primarily happens during sleep. You won't see good results if you don't sleep well.
29. There is no single “best” plan. It's okay to slack off when you’re stressed, and the same applies to fitness. Learn to adjust; exercise is not your entire life. Someone else's training plan may not be suitable for you. Learn to be discerning. It's more meaningful to find someone with similar goals or body type to use as a reference.
30. The look of “fat-wrapped muscle” is a normal part of human anatomy. The structure is layered: skin, then fat, then muscle. Many concepts are just marketing hype. There's no need to force yourself into these boxes. If you need to lose fat, lose fat. If you need to build muscle, build muscle. Live a little more freely.
31. There are many options for cardio after your weight training besides running. You can set the treadmill to an incline and power walk, or use the stair climber. Pay more attention to your heart rate than how out of breath you feel.
32. “Overtraining” typically only happens to advanced and professional athletes. For most people, your body has a limit where it will activate a protective mechanism and tell you “no way”, a point you can’t push past with willpower alone. As a beginner, you don't need complex recovery methods for fatigue management. If you're tired, rest. But it's normal to have muscle soreness 1-2 days after a workout. Rhabdomyolysis is caused by doing excessively intense training after a long period of inactivity.

To be continued……(Women Fitness Administrator, 2022c)

Violations Log (Post Violator’s Profile Link in this Thread)

Must-read for new members: community guidelines, community’s mission, and helpful tips and guides for workouts.

The banned content includes:

1. Insults, body-shaming, and male-validation discourse.
2. Posts promoting extremely low body fat or body anxiety.
3. Redundant questions, low-effort/spammy content.
4. Photo-only posts without privacy protection or context.
5. Multiple check-in threads by the same account.
6. Advertising, irrelevant links, and non-fitness content.

Violations trigger strict enforcement. First time results in post blocking, while repeated violations lead to membership revocation. (Women Fitness Administrator, 2022d)

Prohibited Words Suggestion Thread

Terms related to slut-shaming, pornography, objectification, or unrealistic beauty standards are not permitted in Women Fitness. This includes phrases such as “90-degree shoulders” “A4 waist” “barrel waist” “mom hips” “H-shaped waist”, and references to “weight loss”. (Women Fitness Administrator, 2022e)

Must-read for Beginners: Women Fitness’s Highlight Posts

- Foundational Knowledge about Fitness

“Must-Read Before Starting Strength Training for Absolute Beginners”

“123 Fitness Facts You Should Know (Continuously Updated in Comments)”

“Rather Than Going to the Hospital for Body Fat Testing, Try This...”

“Common Questions About Fat Loss”

“Understanding the Caloric Deficit in Fat Loss”

“All About Glutes – Q&A Compilation v2”

“How to Transition from Training with a Personal Trainer to Training Solo”
“Basic Principles and Methods of Strength Training”

“Share Your Fitness Tips to Help Beginners Avoid Pitfalls (Continuously Updated)”

“To Those Unsure If Their Workout Plan Makes Sense”

“Waist Trainers Are the Foot-Binding of Europe; Lifting Belts Are for Healvy Training”

“One Year Training from Zero: 4.4 Muscle Gain (With Tips)”

“Beginners Should Avoid Using the Smith Machine”

“Is Core Equal to Abs? Can Everyone Get a Six-Pack?”

- Training Mindset and Body Image

“Pull-Up Achievers, Please Join the Hall of Honor”

“Reduce Aesthetic-Centric Workout as Much as Possible”

“Fitness in Everyday Life: Lifting 2000+ Kilograms of Grapes in One Day”

“A Real Body Fat Percentage Story – Say Goodbye to Body Image Anxiety”

“Middle-Age Fitness Wisdom: Life-Long Exercise Beyond the Gym”

“Recommended Video: Reject Body Image Anxiety, Resist Unrealistic Beauty Standards”

“Changing My Fitness Mindset Helped Me Confront More in Life – Strength Gives Confidence”

“Train with a Female-Centered Mindset: Fitness for Freedom, Not for Beauty”

“About ‘Fat Covering Muscle’”

“Causes and Prevention of Osteoporosis”

- Nutrition and Diet

“How Beginners Should Manage Nutrition at the Gym – Copr. Good Person Songsong From Bilibili”

“Beware of Diet Myths – Carbs and Protein Are Equally Important!”

“Fitness Beginners’ Guide to Diet – Part 2”

- About Whether Hiring a Personal Trainer

“My Personal Experience with Hiring a Coach”

“A Universal Standard for Picking a Good Personal Trainer”

- Training During Menstruation

“The Queen of Women’s Fitness Topics - Menstruation”

- Fitness Equipment

“Training Shoes Recommendations (Strength Training, Group Classes, CrossFit)”

“Fitness Clothing Thread”

“Long-Distance Running Gear”

- Injury and Recovery

“Experienced Retinal Detachment – High Myopia Needs to Be Extra Cautious with Weight Lifting”

“Corrective Training (Lots of Images)”

“Hidden Factors Affecting Squats – Outward Toes”

“Rehabilitation Journey from Lumbar Disc Bulge”

“Two and a Half Months of Ankle Sprain Recovery”
“Understanding Shoulder Clicking and Pain”

- Specialized Training

“Self-Taught Swimming Over 2 Years – Reached the Level of a Class 3 Swimmer (With Methods and Videos)”

“Brazilian Jiu-Jitsu: The Best 1v1 Self-Defense Skill for Women”

“Archery First-Time Experience Log”

“Six Months of Boxing Experience”

“Aerobic Capacity Testing”

“National Day Holiday Assignment: Breathing and Core”

“I Fought in a Boxing Match”

“First-Half Marathon Completed – Running Tips”

“Weight-Lifting Girl Climbs 21Montains in a Year”

“Exploring New Training Modes: for Physicality, Cardiopulmonary, and Superb Happiness”

“Rugby for Beginners – Female-centered Guide”

“My Caroline Workout Experience Summary (Muscle Gain Bible) Updated 20240908”

“1.5 Years of Hiking – Saw So Many Landscape”

“Perfect Squat Trajectory – Super Happy!”

“Lessons from My Boxing Gym”

“Hydrophobia Overcome – Learned to Swim in a Week with Nose Clip”

“About On-Ice and Off-Ice Figure Skating Training”

“Any Strong Women Who Play Tennis? Tips Wanted”

“How to Teach a Non-Swimmer — Swimming Water Acclimation Steps and Methods”

“Only Asian in the Group – Went to France for a Training Camp!”

“When Archery, People Become Single-Minded! (Updated with Tips from Sisters Outside Our Community for Those Who Want to Start Archery)”

“How I Did My First One-Arm Pull-Up”

“Boxing Changed Me – Reflections and Joy [Share + Recommend]”

“The Infinite Fun of Combat Sports – A Journey of Cultivating Wisdom and Courage”

“Poor People's Outdoor Guide. Did Some Research on Multi-Use Helmets for Skateboarding/Skiing/Mountain Biking [Finished Update]”

“Sharing Bouldering Beginner Entry and Advanced Techniques”

“Bouldering Cured My Body Image Anxiety”

“Push-Up Tutorial for Absolute Beginners”

“"Highly Recommend Boxing to Everyone!”

“Explaining Weightlifting, Powerlifting, Bodybuilding, CrossFit – The Differences”

“Powerlifting Enthusiast Talks About the Standards for the Big Three Lifts”

- Enhancing Personal Knowledge

“How to Stay Fit and Energetic into Your 100s (Notes from *Outlive*”

“Must-Read Books for Strength Training [Purely Sharing]”

“Basic Human Skeletal Anatomy”

“Basic Muscle Anatomy Diagram”

“[Updated to Part 10 – Core] Reading Notes from *The Women's Fitness Book*”

“Intro Methods I Recommend + Fitness Knowledge Influencers”

- Home Gym Setup

“Some Reference for Those Who Want to Lift Weights at Home, My Mini Home Gym Building History (Updated 23.06)”

“Happy Weight Loss | Home Exercise | Mindset Adjustment | Course Recommendation | 900 Days of Fitness Log”

“How to Choose Tools for Home Training”

- Inspirational & Empowering Content “”

“Should Women Carry Weapons for Self-Defense?”

“From Abandoned Baby to MMA Prodigy: The Power and Possibilities of Women”

“The Joy of Lifting (Updated July 14)”

“Women Are Not Physically Weak”

“I've Got Bicep Separation”

“Because My Pecs Are Big, Men Constantly Tell Me I've Lost My Breasts (Update)”

“From Hating Exercise to Craving It Over 4 Years (A Bit Long)”

“Reduced 18.5kg and Then Gained Muscle, Changes from 61kg to 42.5kg and Then 45kg”

- Supplements

“Personal Review of Various Protein Powder Flavors + Nutrition Facts (Panda/Oxygen/ON)”

“Subjective Reviews of 4 Protein Powders + General Info”

- Advanced Training

“How to Improve VO2 Max [Practical Operations]”

“About [Rigidity]”

“Human Foundation—Feet [Part 1-3]”

“BFRT(Blood Flow Restriction Training) Assistance Methods”

“Fatigue Management Course: What is Fatigue [Part 1]”

“Fatigue Management Course [Part 2] How to Manage Fatigue”

“Fatigue Management Course [Part 3] Fatigue and Overtraining”

“Knowledge Expansion - Factors Affecting Strength [Advanced Topic for Filling Gaps]”

“Often Ignored But Crucial – Training Tempo”

- Caring for Parents

“Exercise Guide for Middle-Aged and Seniors [Study Post]”

“Causes and Prevention of Osteoporosis”

- Community Guidelines

“What to Do If You’re Harassed via DM”

“Guidelines Additions on Controversial Posts About Low Body Fat & Body Image Anxiety Discussions”

“Guide for Sisters Applying to Join and New Members”

“Fast-Track Entry Option”

Community Guidelines and Membership Updates

Recently, certain events within other female-only communities have come to our attention. We have noticed several newly registered users, especially with momo avatar with no posting history on their homepage, are attempting to disrupt discussions across various female-only communities.

Their common tactic involves deliberately misinterpreting discussions within the female-only communities with twisted narratives and false accusations, stirring up emotions among those who don’t know the context. Following this, they will suggest that the community leader and administrative team are problematic or biased. Simultaneously, a set of accounts, also newly registered, will appear to echo these accusations, escalating the situation to a new level.

These accounts operate as a coordinated organization, waiting for opportunities to incite negative emotions or initiate polls. They often come with a set of new accounts and comment control tactics, appearing exceptionally active when needed, posting similar content frequently in short bursts. Women Fitness is also one of their targets,

Community Guidelines Update: Any posts unrelated to fitness/exercise, or those intended to provoke trouble or incite flame wars, will result in immediate removal from the group without warning. This rule is effective immediately.

Entry Filter: No new members will be admitted for the next two weeks, effective immediately.

Starting 8 August, accounts with fewer than ten posts on their profile page will not be approved. (Profile screening for new members will be stricter to prevent applicants from faking being female) (Women Fitness Administrator, 2023)

A Fast-Track Admission Channel is Now Available

In light of the constructive atmosphere within Women Fitness, and in order to promote the exchange of specialized sports knowledge within our community, a fast-track admission has been introduced. Applicants who can provide verifiable evidence for their womanhood, along with the documentation of professional experience or expertise in athletic disciplines, and who are willing to contribute by sharing their knowledge through posts, may request expedited review by directly contacting the administrators. (Women Fitness Administrator, 2022a).

References

*关于最近组内追求低体脂与身材焦虑等具有争议帖子的组规增项*. https://www.douban.com/group/topic/271672958/?_i=6494753GB1us8m

Women Fitness Administrator. (2022b, July 16). *日常举报组务专楼【举报用，勿回】*. https://www.douban.com/group/topic/270944138/?_spm_id=NjM1OTgyMzg&_i=0242911LE_IwtM

Women Fitness Administrator. (2022c, July 27). *说说你们知道的健身小tips，帮助新人小白少走弯路（评论整理持续更新）*. https://www.douban.com/group/topic/271679859/?_i=0242842LE_IwtM

Women Fitness Administrator. (2022d, August 29). *违规记录楼（组内违规粘贴主页进此楼）*. https://www.douban.com/group/topic/273961155/?_i=0242770LE_IwtM

Women Fitness Administrator. (2022e, October 25). *违禁词设置建议楼*. https://www.douban.com/group/topic/277394255/?_spm_id=MjU0MzM1MTk1&_i=0404569LE_IwtM

Women Fitness Administrator. (2023, July 24). *组规＆举报 新增一条组规⚠️*. https://www.douban.com/group/topic/292211093/?_spm_id=MjU0MzM1MTk1&_i=0231688LE_IwtM
